# Top-down mass spectrometry of native proteoforms and their complexes: A community study

**DOI:** 10.21203/rs.3.rs-3228472/v1

**Published:** 2023-08-23

**Authors:** Frederik Lermyte, Tanja Habeck, Kyle Brown, Benjamin Des Soye, Carter Lantz, Mowei Zhou, Novera Alam, Md Amin Hossain, Wonhyeuk Jung, James Keener, Michael Volny, Jesse Wilson, Yujia Ying, Jeffrey Agar, Paul Danis, Ying Ge, Neil Kelleher, Huilin Li, Joseph Loo, Michael Marty, Ljiljana Pasa-Tolic, Wendy Sandoval

**Affiliations:** Technische Universität Darmstadt; Technische Universität Darmstadt; University of Wisconsin-Madison; Northwestern University, Illinois, USA; University of California, Los Angeles; Pacific Northwest National Laboratory; Northeastern University; Northeastern University; University of California, Los Angeles; University of Arizona; Genentech Inc.; Pacific Northwest National Laboratory; Sun Yat‐sen University; Department of Chemistry and Chemical Biology, Northeastern University; Eastwoods Consulting; University of Wisconsin-Madison; Northwestern University; Sun Yat‐sen University; University of California, Los Angeles; University of Oxford; Pacific Northwest National Laboratory; Genentech Inc.

## Abstract

The combination of native electrospray ionisation with top-down fragmentation in mass spectrometry allows simultaneous determination of the stoichiometry of noncovalent complexes and identification of their component proteoforms and co-factors. While this approach is powerful, both native mass spectrometry and top-down mass spectrometry are not yet well standardised, and only a limited number of laboratories regularly carry out this type of research. To address this challenge, the Consortium for Top-Down Proteomics (CTDP) initiated a study to develop and test protocols for native mass spectrometry combined with top-down fragmentation of proteins and protein complexes across eleven instruments in nine laboratories. The outcomes are summarised in this report to provide robust benchmarks and a valuable entry point for the scientific community.

## Introduction

A single gene can give rise to many distinct proteoforms.^1–3^ The need to accurately identify and structurally characterise these proteoforms has led to significant technological developments in recent years, most notably in so-called ‘top-down’ mass spectrometry (MS) of intact proteins.^4–9^ This term refers to measurement of the intact mass of the protein, followed by gas-phase fragmentation to obtain sequence information. In 2021, the Human Proteoform Project was conceived, with the ambitious goal of producing a high-quality atlas of human proteoforms.^10^ However, in addition to the precise primary structure of a proteoform, it is also critical to characterise its interactions and corresponding noncovalently bound functional complexes with other proteins and/or co-factors. This information is lost in most top-down MS experiments, which are carried out under denaturing solution conditions. Conversely, ‘native’ MS, in which noncovalent complexes are preserved during transfer into the gas phase, offers a powerful method to characterise interactions between proteins and to determine the stoichiometry of complexes.^11–15^ The power of the combination of native ionisation with extensive top-down proteoform characterisation has been convincingly demonstrated in recent years, for example in the identification of novel endogenous complexes, previously unknown co-factors, and regulatory post-translational modifications.^16–20^ However, the use of this powerful integrated approach is still rare.

**Figure 1:**
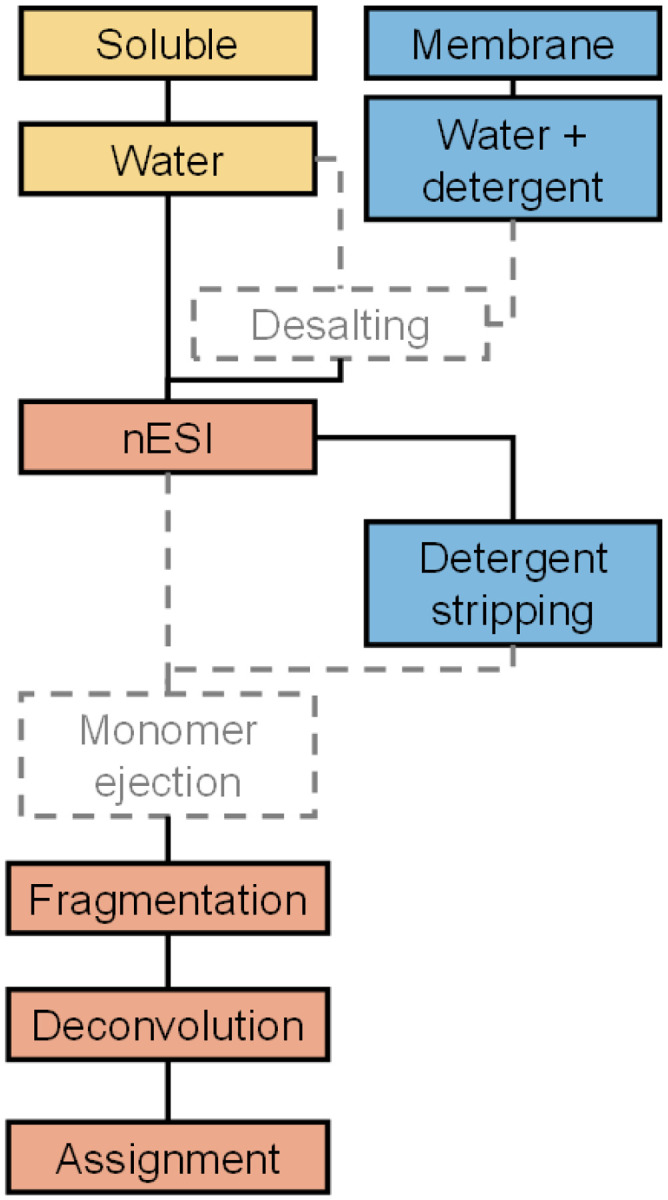
Summary of the protocol for top-down analysis of natively ionised proteins used by study participants. Optional steps are shown in dashed lines, steps specific to water-soluble proteins in gold, and those specific to membrane proteins in blue.

The limited adoption of this approach, known as ‘native top-down’,^21^ ‘nativeomics’,^18^ and ‘complex-down’^22–25^, is primarily due to the perception that both native and top-down MS are challenging, let alone the combination of the two. Although efforts have been made in recent years to make both methods more widely applied,^4,6,7,26–28^ neither has been standardised to the same extent as conventional bottom-up proteomics. Furthermore, even if one overcomes the barrier to entry that is created by the lack of standardisation and successfully carries out the different steps of this type of experiment, *i.e*., native ionisation (with electrospray ionisation), gas-phase disassembly of the complex, subsequent backbone fragmentation, and analysis of the resulting data (see Fig. 1), there is still the issue of assessing the quality of the results. The relatively sparse relevant literature makes it difficult for a newcomer to the field to identify appropriate benchmark data, ideally acquired on a similar instrument with a protein that is conveniently available.

Having identified this unmet need for robust protocols and benchmarks, the Consortium for Top-Down Proteomics (CTDP) launched the ‘Native Top-Down Initiative’ in 2020. A range of laboratories, covering all major instrument families for protein MS, were invited to perform native ionisation with subsequent top-down fragmentation of a set of six standard proteins that covered a broad mass range from 27 to 800 kDa. These included monomeric as well as multimeric proteins, and water-soluble as well as membrane proteins. It is important to note that not all participants had prior experience with this type of experiment; therefore, the protocols used in this study can demonstrably be implemented by users with various levels of expertise. This report will convincingly lower the entry bar and serve as a convenient benchmark.

## Results

### Study design

Nine laboratories generated data in this study, with a total of eleven instruments: 5 Orbitraps, 4 quadrupole/time-of-flight (QTOF), and 2 Fourier transform ion cyclotron resonance (FTICR) instruments. At the beginning of the study, before any data collection was performed, five of the participants collaboratively created an optimised protocol which was then used by all nine participants. A visual summary of this protocol is shown in Fig. 1, and an overview of the model proteins and acquired datasets is shown in Table 1. The protocol itself can be found in the **Supplementary Information**. Participants were allowed free choice of the methods for spectrum deconvolution and fragment assignment. The methods they used are summarised in **Supplementary Figure S1**. For ion activation and fragmentation, they were instructed to use only collision-based methods to facilitate comparison between datasets. At the start of the study, participants were asked to self-report their experience level with native (top-down) MS. At the end, they were asked to self-report the perceived difficulty of different steps of the experiment. An overview of these survey results can be found in **Supplementary Figures S1 and S2**. Overall, the responses indicated a relatively broad range of experience levels at the start of the study, as well as a consensus about the relative difficulty of most of the analysis steps, with obtaining extensive backbone fragmentation and analysis of membrane proteins being particularly challenging.

### Similar native spectra across different laboratories and instruments

**Figure 2:**
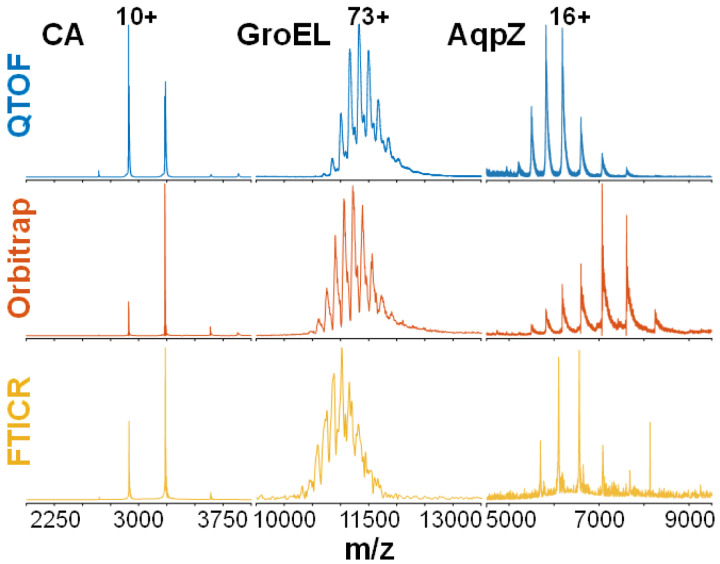
Native mass spectra of **(a)** CA, **(b)** GroEL, and **(c)** AqpZ (detergent: C8E4), acquired on FTICR, QTOF, and Orbitrap instruments (data from 5 participating laboratories in total). Additional native spectra can be found in **Supplementary Figure S3**.

Before addressing the fragmentation data, we wished to benchmark the native MS experiment itself. There have been previous efforts to accomplish this;^6,27^ however, here we had access to a much larger dataset, with more proteins, more instruments, and more laboratories. To our knowledge, our work represents the first multi-laboratory study of both native MS and native top-down MS of a range of protein complexes. In the literature, there is broad consensus that native MS leads to lower charge states and a narrower charge state distribution compared to MS under denaturing conditions.^11,29^ There have been previous efforts to study the relationship between protein mass, conformation, and charge state;^29–32^ however, these studies have typically been carried out by single laboratories, and as such, it is unclear to what extent the observed native spectra depend on instrument- or operator-related factors. Figure 2 shows representative native spectra for three proteins from our set of samples, each acquired on three different instrument types. It is immediately apparent in this figure that the native charge state distributions are consistent between different operators and instruments, indicating that this property is indeed mostly determined by protein-specific factors. This consistency also suggests that the different nano-electrospray sources produced ions with similar conformations and internal energies. Additionally, the correct stoichiometries were observed for the oligomeric species with different instruments, which supports the increasing adoption of native mass spectrometry as a versatile tool to determine protein complex stoichiometry. Peak width in the native spectra, however, differed significantly between instruments.

### Resolving power and cleavage coverage

**Figure 3:**
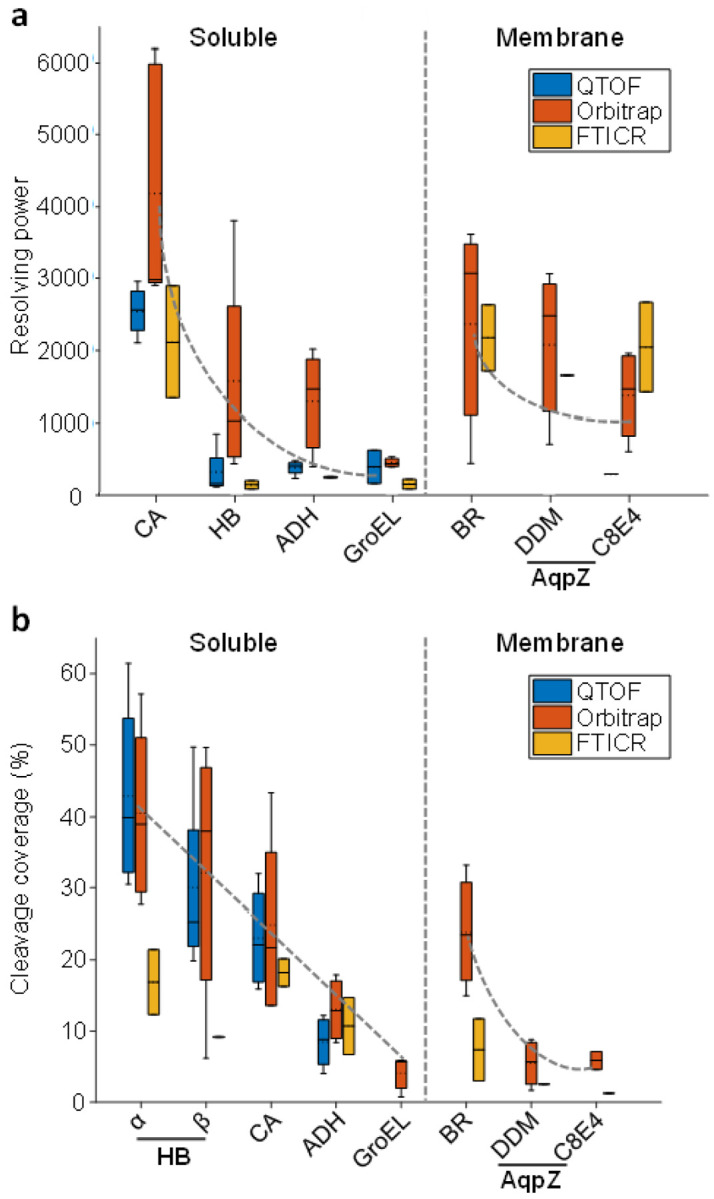
**(a)** Boxplots of the obtained resolving power by protein and instrument type. Proteins are divided into soluble and membrane proteins, and then arranged by increasing precursor mass. **(b)** Boxplots of the obtained cleavage coverage by protein and instrument type. Proteins are again divided into soluble and membrane proteins, but now sorted by increasing monomer mass. Grey dashed lines in both panels are added to guide the eye and show the trend for lower resolving power and cleavage coverage *versus* mass.

For an accurate determination of the mass of a protein, resolving power – which is inversely correlated with peak width – is an important factor. In native MS, significant peak broadening is caused by the formation of nonspecific adducts with water molecules, sodium cations, *etc*. As a result, the effective resolving power (*i.e*., peak centre *m/z* divided by the full width at half maximum) can be orders of magnitude lower than the limit imposed by the mass analyser, and the ability to reduce the presence of the aforementioned clusters is a critical factor. This can be accomplished by thorough desalting prior to ionisation, controlled gas-phase activation for improved desolvation, or a combination of both. Figure 3a shows the effective resolving power obtained for each protein, broken down by instrument type. A clear trend for lower effective resolving power can be seen as the mass of the intact protein ion increases, indicating – as one might expect – that larger proteins tend to retain more water and/or salt during ionisation. We note that the theoretical resolving power limit imposed by the mass analyser decreases with *m/z* in Orbitrap and FTICR instruments. In practice, this factor adds only a minor contribution to the observed peak broadening compared to adduct formation.^33^ The type of instrument used still plays an important role, as Orbitrap platforms tended to generate narrower peaks than other instrument types, particularly for water-soluble proteins; however, this seems to be due to differences in desolvation prior to entry in the mass analyser. In some datasets, particularly those acquired with FTICR instruments, the obtained effective resolving power was higher for the membrane protein aquaporin Z compared to soluble proteins of similar mass (haemoglobin and ADH). A possible explanation is that participants erred on the side of using very ‘soft’ instrument settings for water-soluble proteins, which prevented unfolding, but also led to incomplete desolvation. The deliberate harsher tuning required for stripping detergent molecules from membrane proteins therefore may have also improved the removal of water and salt. However, two participants reported the observation of trimeric AqpZ – almost certainly formed through unintentional gas-phase dissociation of the complex during detergent removal – when using DDM to assist in solubilisation rather than the expected tetramer, illustrating the risk of over-activation. Conversely, the C8E4 detergent seems to be easier to remove, providing a greater ‘safety margin’ between detergent removal and disruption of the native complex.^34^

While good resolving power and accurate precursor mass determination are important, a key to proteoform characterisation is the effective generation and identification of backbone fragments. To compare this factor between datasets, we calculated the cleavage coverage - defined here as *the number of observed specific backbone cleavage sites divided by the total number of inter-residue bonds* – as a global metric for each spectrum. We then plotted the cleavage coverage for each protein by instrument type, and this is shown in Fig. 3b (average values across all datasets are reported in Table 1). As was observed for resolving power, a decrease in cleavage coverage with higher mass was apparent. Generally, no instrument type clearly outperformed the others in terms of obtained cleavage coverage. The exception to this was GroEL, for which only Orbitrap instruments, specifically ‘Q Exactive UHMR’ models optimised for the analysis of high-mass ions, yielded interpretable fragment spectra. Representative fragment spectra can be found in **Supplementary Figure S4**.

### Preference for specific fragmentation sites

**Figure 4:**
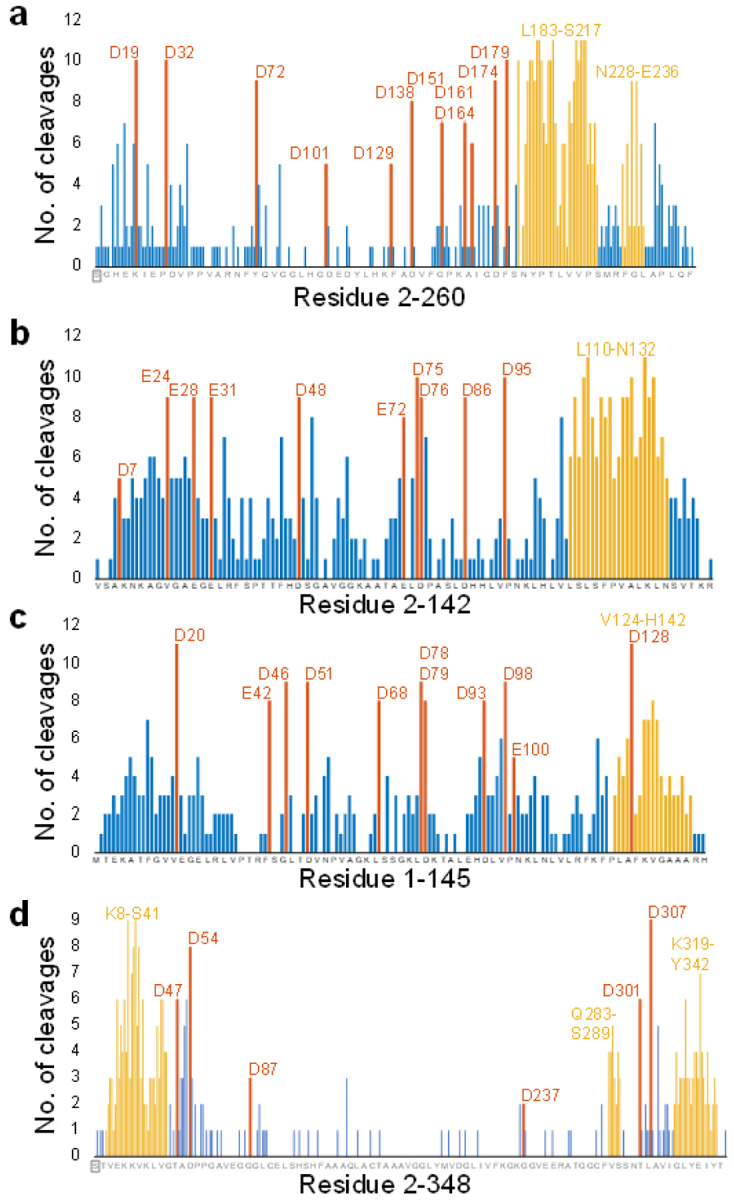
Number of observations of site-specific cleavage in **(a)** carbonic anhydrase, **(b)** alpha subunit and **(c)** beta subunit of haemoglobin, and **(d)** alcohol dehydrogenase. The residue-specific cleavage sites are highlighted in orange, and the most common cleavage regions in yellow. N-terminal acetylation of CA and ADH is indicated with a grey box. Equivalent figures for GroEL, bacteriorhodopsin, and aquaporin Z can be found in **Supplementary Figure S5**.

In addition to global cleavage coverage, the site-selectivity of backbone cleavages is important. Three alternative hypotheses can be postulated *a priori*: (1) cleavage sites are randomly distributed along the protein sequence; (2) there is a systematic, protein-dependent preference for certain cleavage sites; or (3) site preferences are instrument-dependent. In particular, for collision-based dissociation of native proteins on ‘Q Exactive’ Orbitrap instruments, site-selectivity has previously been linked to both residue-specific factors and to higher-order structure, depending on the specifics of the experiment.^35,36^ Here, however, we once again had the opportunity to extend the previous work by including results from different instrument types. The histograms in Fig. 4 show the number of times a cleavage site was observed for carbonic anhydrase, haemoglobin, and ADH, ranging from 0 (fragments resulting from this cleavage were never observed) to 11 (fragments resulting from this cleavage were observed by all labs, with all instruments). Equivalent figures for GroEL, bacteriorhodopsin, and aquaporin Z can be found in **Supplementary Figure S5**. Clear fragmentation ‘hotspots’ can be distinguished, and these are often associated with specific residue types, most notably C-terminal to aspartic acid, as indicated in the plots, and consistent with previous work.^35^ This charge-remote fragmentation pathway is not dependent on mobile protons,^37^ explaining why it is common in the rather low-charge state ions created in native ESI. For the membrane proteins bacteriorhodopsin and aquaporin Z, fragmentation was preferentially observed in the transmembrane helices, in agreement with earlier work.^38^ Remarkably, some fragments were observed in 11 out of 11 datasets, indicating very high inter-laboratory reproducibility, and clearly showing that protein-specific factors are key for determining site-selectivity of fragmentation, regardless of the specific instrument used.

Another interesting pattern visible in both Fig. 3b and Fig. 4 is the greater propensity for fragmentation of the alpha subunit of haemoglobin than the beta subunit. Looking at the overall cleavage coverage values across all datasets, these were (37.1 ± 14.4)% and (27.2 ± 15.6)% for the alpha and beta subunit, respectively. While these confidence intervals do overlap (p = 0.13), it is noteworthy that in 10 out of 11 individual datasets, the cleavage coverage for the alpha subunit was higher, strongly suggesting that these observations indeed reflect an inherent difference in fragmentation propensity. From the cleavage site histograms in Fig. 4, it is apparent that the main C-terminal fragmentation ‘hotspot’ in the alpha subunit extends further toward the N-terminus compared to the equivalent in the beta subunit, and it also contains significantly more sites where cleavage was observed in 10 or even 11 out of 11 datasets. This can be rationalised by comparing the sequence of both proteins, as in the alpha subunit this 30-residue region contains two proline and two aspartic acid residues, as opposed to one of each in the beta subunit. These regions are highlighted in the monomer structures in **Supplementary Figure S6**.

We also performed a small-scale comparison between the obtained cleavage coverage with natively ionised *versus* denatured precursors. For this, three participants, covering all three instrument types (Orbitrap, QTOF, and FTICR), performed top-down CID of denatured carbonic anhydrase and ADH. We selected these specific proteins for two reasons: (1) We already had datasets from all participating labs for each of them under native conditions, and (2) one is a monomer, while the other natively forms a tetramer. During dissociation after native ionisation, the latter ejects a highly-charged monomer, which subsequently fragments, whereas the former can only (partially or fully) unfold and then fragment. For the natively ionised CA and ADH, the average cleavage coverage values achieved by these three participants were (23.7 ± 7.0)% and (10.8 ± 4.0)%, respectively, *i.e*., in line with the (23.5 ± 9.3)% and (10.6 ± 4.5)% across datasets from all labs. With denatured precursors, the corresponding values were (25.0 ±5.5)% and (8.8 ± 4.5)%. While our dataset is too small to draw general conclusions about native *vs*. denatured mode MS, these results indicate – perhaps surprisingly – that at least in CID-based top-down MS of ADH and CA, similar cleavage coverage values can be obtained from natively ionised as from denatured precursors. Charge-remote cleavage C-terminal to aspartic acid was less pronounced for the denatured precursors (see **Supplementary Figure S7**).

### Data analysis in top-down protein mass spectrometry

Software tools used by participants in the analysis of precursor and fragment spectra are summarised in the Methods (Table 2). As MS measures mass-to-charge ratios, the charge states of ions must be determined to assign their mass. This process is quite different for a large, native-like precursor, compared to fragments that often have masses below 10 kDa, and both operations need to be carried out successfully. For fragments, modern MS instruments can often resolve the isotopic distribution, unlike for intact (native) proteins. As isotope peaks are spaced approximately 1 Da apart, measuring the spacing on the *m/z* axis often makes fragment charge state determination trivial. Algorithms that model the isotopic distribution based on the average composition of proteins can then estimate the monoisotopic mass of the fragment for comparing to candidate assignments, or the entire isotopic distribution can be matched to an *in silico* generated one.^39–43^ In contrast, for intact protein precursors, conventional instruments are unable to resolve isotope peaks, and other algorithms for charge state deconvolution are required (*vide infra*).

**Figure 5:**
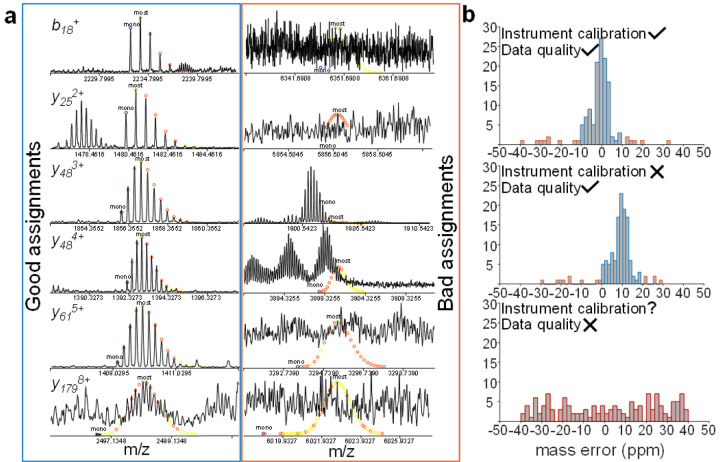
Fragment assignment and data quality assessment. **(a)** Fragment assignments after eTHRASH deconvolution in the MASH software. The blue box shows correct assignments for a range of charge states (coloured dots indicate the calculated isotope distributions). In the red box, incorrect automatically assigned fragments (the result of choosing an inappropriately low signal-to-noise threshold) are shown. Here, either manual rejection in combination with mass error filtering is needed to exclude these fragments, or reprocessing with a higher signal-to-noise threshold. **(b)** Fragment mass error distributions can be used to assess data quality.^57,58^ For real fragment signals, a narrow mass error spread around zero ppm is expected (top panel; blue bars). Poor instrument calibration (middle) shifts the centre of this narrow distribution. Depending on the instrument, narrower distributions (e.g., ±2 – 5 ppm) might be expected. If data quality is poor and mainly noise is assigned (a strong indication of an unsuccessful experiment), evenly distributed error values over a broad range (bottom) are observed.

A particular challenge in top-down MS is that the total signal intensity is divided over a large number of fragments.^44^ Therefore, fragment signal-to-noise ratios can be rather low, and spectral averaging is often required. This procedure usually works well in vendor-specific software; however, freely available alternatives that work with vendor-neutral MS data formats such as mzML often do not perform as well as the proprietary software in our experience. An option that was used by a plurality of participants in this study was the MASH software series,^45–49^ which can read in data from a relatively broad range of instruments, as well as generic formats that can be generated after spectral averaging using vendor-specific software. In top-down MS data analysis, it is important to carefully choose the minimum signal-to-noise level and mass error tolerance. Making these parameters too ‘loose’ will result in many false positive assignments, while making them too restrictive will lead to real fragment signals not being identified (*i.e*., false negatives). Figure 5 illustrates this concept, as well as the use of mass error distributions to assess the quality of fragment assignments. Visual comparison of the calculated (coloured circles) and observed (black solid traces) isotope distributions often makes it obvious whether an assignment is reliable, although the (correct) assignment of a *y*_*179*_^*8+*^ fragment is worth highlighting. This ion had a mass of nearly 20 kDa; therefore, its intensity was distributed over a large number of isotopologues, which led to a reduced signal-to-noise ratio, illustrating the particular risk of overly restrictive search settings in top-down protein analysis.

In contrast to fragment signals, obtaining isotopic resolution is near-impossible for large, native-like species, as peak broadening due to noncovalent adducts limits resolving power values to a few thousand at best (as shown in Fig. 3a). Different deconvolution strategies are thus required for precursors than fragments. Charge detection approaches allow direct measurement of an ion’s charge state;^50–53^ however, this is not (yet) common and in practice it is usually necessary to observe multiple successive charge states and then apply a probabilistic deconvolution algorithm. While several algorithms and software packages exist, a majority of participants in the current study used the freely available UniDec software^54–56^ for deconvolution of precursor spectra (see **Supplementary Figure S1**). This software provides a mass and intensity list for the main ion series as well as less abundant species, along with a confidence score for each. UniDec is not vendor-specific as a spectrum list can be used as input; however, it also supports upload of raw files from several different instrument manufacturers. UniDec is included in the latest iteration of the MASH software package (MASH Native).^49^

## Discussion

Native and top-down mass spectrometry have each in their own right had a significant impact on our understanding of molecular biology, and the combination of both methods promises to open up new scientific vistas. At the same time, however, newcomers to the field experience significant barriers to entry, and more experienced practitioners regularly receive requests for advice on how to get started. Here, we have developed protocols for this type of experiment and demonstrated their implementation across a range of laboratories and instruments. Our post-study surveys (see **Supplementary Figure S2**) showed that (nano-)ESI and gas-phase activation of membrane proteins were experienced as more challenging than the experiments with water-soluble proteins. The exception is the water-soluble GroEL, for which, as previously mentioned, only high-mass modified Orbitrap instruments were able to induce monomer ejection and extensive backbone fragmentation. This can be explained by the large size and stability of this complex, which results in a high amount of energy being needed for monomer ejection and backbone fragmentation. The high-mass range Orbitraps seem to be more capable of supplying this amount of energy than other, less specialised instrument types.

The protocols described in this study will empower newcomers to the field(s) of native and/or top-down protein MS to successfully ionise monomeric and multimeric, water-soluble and membrane proteins in native mode across a wide mass range, and to obtain backbone fragmentation from these ions. In particular, as native separation methods such as capillary electrophoresis and size-exclusion chromatography are increasingly coupled with top-down approaches, these tools will drive new biological insights through high-throughput analysis of protein complexes. The example data shown in this report, as well as the high-level summaries of obtained resolving power and cleavage coverage per instrument type, provide a valuable benchmark to assess data quality for several easily available protein standards. One potential risk is overreliance on the overall cleavage coverage benchmarks, as in an attempt to reach the target coverage value, newcomers might feel encouraged to inappropriately loosen search parameters in their data analysis. Therefore, these numbers should be used together with the cleavage site frequency data: If similar site-selectivity is observed in conjunction with a similar overall coverage, one can be rather confident that a workflow is reasonably optimised. We also refer to the representative fragment spectra from different instrument types in **Supplementary Figure S4** for further guidance.

The combination of native MS with top-down fragmentation is able to provide uniquely detailed information on proteoform-specific biomolecular interactions. In particular, this combination is not only able to identify and characterise proteoforms, but also to link this information to protein structure, complex stoichiometry, and other noncovalent interaction partners. The benchmarks and guidance provided in this report will lead to a more widespread adoption of these methods by the scientific community in the coming years, so that they can be used to answer important biological questions.

## Methods

The proteins used for this study were either bought from Sigma or (in the case of AqpZ) supplied by one of the participants. More information and catalogue numbers can be found in **Supplementary Table S1**. All participants prepared the protein samples according to the given protocol (see **Supplementary Information**). In general, all soluble proteins were dissolved in 200 mM ammonium acetate solution, and for membrane proteins the respective detergent was added in 2x critical micelle concentration. After desalting, all labs used nano-ESI with slightly different setups. The applied spray voltage ranged from 0.7 to 2 kV depending on the instrument setup and sample. Table 2 shows the used instrument types and data analysis software per lab. More details can be found in **Supplementary Table S2**, including important tuning parameters for alcohol dehydrogenase (provided as an example and potential starting point) on all instruments used.

## Figures and Tables

**Table 1.** Overview of the datasets generated in this study (note: the mass value in the second column is for the native multimer, where applicable). Proteins in the first four rows are water-soluble; those in the last three are membrane proteins. More information on these proteins can be found in **Supplementary Table S1**.

Protein	Mass (kDa)	Subunits	Datasets	Average obtained cleavage coverage	Typical native precursor charge state for activation
**Soluble proteins**					
Carbonic anhydrase 2 (CA)	29	1	11	(23.5 ± 9.3)%	10 +
Haemoglobin (HB)	64	4	11	a: (37.1 ± 14.4)% b: (27.2 ± 15.6)%	16 +
Alcohol dehydrogenase (ADH)	148	4	10	(10.6 ± 4.5)%	25 +
GroEL	801	14	3*	(4.1 ± 2.4)%	70 +
**Membrane proteins**					
Bacteriorhodopsin	27	1	5	(17.3 ± 10.3)%	9 +
Aquaporin Z (AqpZ) (detergent: C8E4)	99	4	3**	(4.3 ± 2.4)%	16 +
Aquaporin Z (AqpZ) (detergent: DDM)	99	4	5***	(4.9 ± 2.9)%	16 +

* In total 7 participants carried out native MS of GroEL; however, only 3 (all using Orbitrap UHMR instruments) generated interpretable fragment spectra from this precursor;

** 7 participants successfully carried out native MS of AqpZ in C8E4; however, only 3 (2 with Orbitrap UHMRs, 1 with an FTICR) were able to generate interpretable fragment spectra;

*** of the 5 datasets with fragment spectra, only 4 reported observation of the tetramer (3 Orbitrap UHMRs, 1 FTICR), while the fifth reported a trimer. A sixth dataset (acquired on a QTOF) reported trimeric AqpZ, but fragmentation was unsuccessful.

**Table 2:** Instruments and software used by each participating lab. Software used for precursor and fragment spectra are indicated by * and †, respectively.

Lab	MS instrument	Data analysis software
1	Waters Synapt G2Si	UniDec*, LcMsSpectator†
2	Orbitrap Q-Exactive UHMR	UniDec*, MASH Explorer†
3	Bruker solariX FTICR	DataAnalysis*, MASH Explorer†
4	Waters Synapt G2Si	MassLynx (manual data analysis)*†, UniDec*
5a	Bruker solariX FTICR	DataAnalysis*† ClipsMS†
5b	Orbitrap Q-Exactive UHMR	BioPharma Finder*†, ClipsMS†
6	Orbitrap Q-Exactive UHMR	UniDec*, TDValidator†
7	Orbitrap Q-Exactive UHMR	UniDec*, FreeStyle Extract†, ProSight Lite†
8a	Waters Synapt XS	UniDec*, MASH Explorer†
8b	LTQ Orbitrap XL	UniDec*, MASH Explorer†
9	Agilent 6545XT SLIM QtoF	Agilent Bioconfirm 10*†, deCharger†, MASCOT†, ProSight Lite†
